# Association of the IL-4R Q576R Polymorphism with Pediatric Asthma: a meta-analysis

**DOI:** 10.4314/ahs.v22i3.32

**Published:** 2022-09

**Authors:** Xiwu Chen, Jinhui Hu, Kaiwei Li, Sheng Liu, Qin Ye, Baocen Cao

**Affiliations:** Department of Pediatrics, Huangshi Central Hospital, Affiliated Hospital of Hubei Polytechnic University, Edong Healthcare Group, Huangshi, Hubei 435000, China

**Keywords:** IL-4, Asthma, Meta-analysis

## Abstract

**Background:**

The relationship between Q576R polymorphism of IL-4 receptor (IL-4R) gene and pediatric asthma risk is still undefined. To this end, this meta-analysis was performed to explore the above controversy.

**Methods:**

In this study, we systemically retrieved CNKI, EMBASE, Web of Science, Scopus, Science direct and Pub Med to collect relevant researches, followed by calculation of odds ratio (OR) along with 95% confidence intervals (CIs). STATA 12.0 software was employed in this meta-analysis.

**Results:**

We found an association between IL-4R Q576R polymorphism and pediatric asthma risk (GG vs AA: OR = 3.75, 95% CI = 1.89–7.45; AG vs AA: OR = 2.15, 95% CI = 1.36–3.39; the dominant model: OR = 2.25, 95% CI = 1.42–3.57;the recessive model: OR = 3.05, 95% CI = 1.54–6.05). Moreover, there was no obvious publication bias.

**Conclusion:**

Our findings suggested that G allele of IL-4R Q576R polymorphism is associated with increased risk of pediatric asthma. Anyhow, delicately-designed, large-scale studies should be conducted to further confirm the current outcomes.

## Introduction

Asthma is a common chronic allergic disorder that involves the respiratory tract and affects millions of children worldwide. With the detection rate up to 10% of children, atopic asthma is a global public health problem^1^. The complex pathogenesis of asthma is still unclarified. However, decreased lung function, allergic disorder, bacterial and viral infections are currently considered as the main causes for the persistence as well as progression of asthma^2^–^6^. Additionally, according to epidemiological researches, there is certain association between asthma etiology and genetic risk factors^7^. Genetic risk factors for adult-onset asthma are largely a subset of the genetic risk for childhood-onset asthma but with overall smaller effects, suggesting a greater role for non-genetic risk factors in adult-onset asthma^8^. Moreover, parental asthma is a potent predictive factor for pediatric asthma, suggesting the potent genetic basis of pediatric asthma^9^.

Interleukin 4 (IL-4) not only is vitally involved in type 2 T-helper (Th2) reactions and isotype class switching of B cells to IgE synthesis, but participates in the recruitment of mast cells^10^. IL-4 receptor (IL-4R) is a transmembrane protein containing two subunits, namely α and γ chains. Moreover, accumulative studies have supported the critical functions of IL-4R in asthma pathogenesis and IgE level regulation. The binding between IL-4 protein and IL-4R triggers tyrosine system activation, which subsequently activates signal transducer and activator of transcription 6 (STAT6), thereby elevating the level of IL-4-sensitive genes, including IgE, MHC-II as well as CD23^11^.

IL-4R gene has been previously demonstrated as a potential asthma-related gene, located on chromosome 16p12.1. IL-4R Q576R polymorphism (rs1801275) was first revealed by Hershey et al. in 1997, which was potently correlated with atopy^12^. IL-4R Q576R polymorphism of exon region gives rise to the glutamine-to-arginine substitution IL-4Rα protein, which is present on the cytoplasmic domain. At present, IL-4R Q576R polymorphism has been demonstrated to be associated with a series of disorders, such as bronchiolitis, periodontitis and atopic dermatitis^13^–^15^.

Further investigations have indicated the possible relationship of IL-4R Q576R polymorphism with pediatric asthma, however, the conclusion is drawn from studies with inadequate statistical power, sample size and clinical heterogeneity. To this end, the present meta-analysis was performed to accurately examine the correlation of IL-4R Q576R polymorphism with pediatric asthma risk.

## Methods

### Identification of studies

Two investigators (Xiwu Chen and Jinhui Hu) screened each of the titles, abstracts and full texts to determine inclusion independently. The results were compared and disagreements were resolved by consensus. We systematically searched in Embase, Pub Med, Web of Science, Scopus, Science direct and CNKI databases with several key words: ‘interleukin 4/IL-4’, ‘576R’, ‘pediatric asthma’, and 'gene polymorphism’ (last search was updated on October 2021). Additional manual screening of references from reviewed studies was supplemented to comprehensively extract all relevant researches. Moreover, an information flowchart was constructed, covering screening, identification, eligibility and final selections based on Preferred Reporting Items for Systematic Reviews and Meta-analyses (PRISMA) guidelines^16^. The protocol is registered in PROSPERO (ID: 283601).

### Inclusion criteria and data extraction

Eligible researches were collected accordingly: i) All studies concerning the association between IL-4R Q576R polymorphism and pediatric asthma risk; ii) researches with adequate and effective genotype information; iii)We defined childhood asthma using strict age of onset criteria and considered asthma cases with onset < 12 years of age as childhood onset cases^8^. Additionally, comparisons of animal study, laboratory approaches, interim analysis and researches with overlapping populations were eliminated.

### Data extraction

Necessary data were retrieved from all eligible researches by two independent investigators with agreement on all items (Xiwu Chen and Jinhui Hu), including: first author name, year of publication, region, number and genotypes of cases and controls, ethnicity and evidence of Hardy-Weinberg equilibrium (HWE) in controls.

### Quality assessment

Newcastle-Ottawa Scale (NOS) was employed to evaluate the methodological quality of every enrolled research^17^, where a final score of or over six stars was taken for high-quality.

### Statistical analysis

The deviation from HWE for the distribution of every genotype in control group was determined by Fisher exact test. In addition, odds ratio (OR) along with 95% confidence interval (95%CI) was adopted to assess the degree of correlation between IL-4R Q576R polymorphism and vulnerability to pediatric asthma under Dominant (GG+-GA vs. AA), Additive (GG vs. AA, GA vs. AA) and Recessive (GG vs. AA+GA) models between groups. I2 test was subsequently employed to evaluate the heterogeneity among researches, where I2 > 50% implicated heterogeneity as well as application of random effects model, otherwise fixed effects model should be performed. Sensitivity analysis was mainly conducted by sequentially omitting each study or non-HWE ones. Finally, the possible publication bias was visually assessed by Begg's funnel plot. STATA 12.0 (Stata Corp LP, College Station, TX, USA) was employed for statistical analysis and a P value <0.05 indicated statistical significance. The power of each study was computed as the probability of detecting an association between IL-4R Q576R polymorphism and pediatric asthma using a significance level of 0.05, assuming an OR of 1.5 (small effect size). Power analysis was per¬formed using the statistical program PS: Power and Sample Size Calculation (http://biostat.mc.vanderbilt.edu/wiki/Main/PowerSampleSize).

### Trial sequential analysis

Meta-analysis might be affected by the increased risk of random errors and repeated significance testing. TSA can increase the robustness of the conclusions by estimating the amount of the required information size (RIS) and the threshold for statistical significance. During the analysis, the significance levels for type I and type II errors were set to 5% and 20%, respectively, and relative risk reduction (RRR) was set at 20%. When the cumulative Z-curve crosses the TSA boundary or enters the insignificance area, it demonstrates a sufficient level of evidence, and no further study is necessary. The TSA software (version 0.9.5.10 beta) was used for data processing^18^.

## Results

### Study characteristics

**Figure 1:**
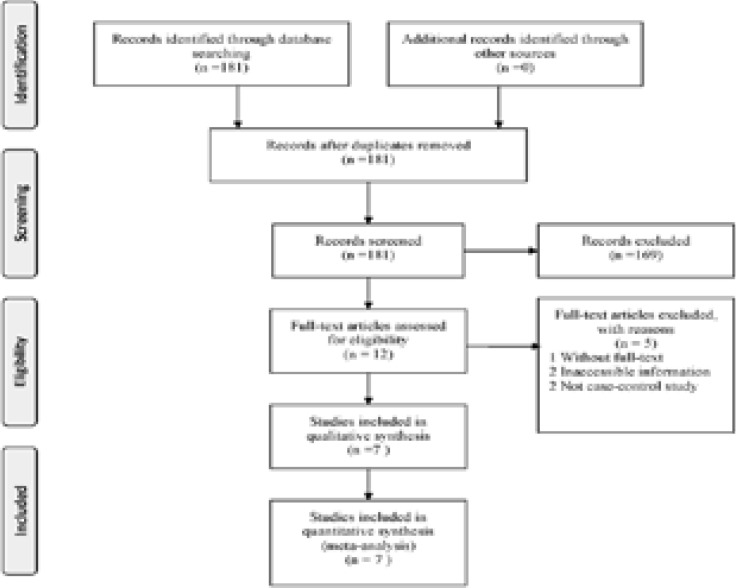
The flow diagram of included/excluded studies.

The flow diagram concerning study selection was displayed in Figure 1. 181 studies were initially identified, and 169 of them were eliminated after reviewing title or abstract. Moreover, one, two and another two studies were eliminated due to no full-text, not case-control studies and inaccessible necessary information, respectively. Eventually, seven researches were enrolled in our meta-analysis, including a total of 912 cases and 708 controls^19^–^25^. Except for the study by Zheng et al., the genotype distribution was consistent with HWE in control populations. All included studies used PCR-RFLP method. The main features of these researches were displayed in Table 1. NOS score of all studies were higher than six stars was taken for high-quality studies. The statistical powers of these 7 studies ranged from 18.2% to 49%. None of the studies had a statistical power that exceeded 80%.

**Table 1:** Characteristics of the included studies for meta-analysis

Study included	Year	Area	Race	Cases/ Controls	Genotypes for cases			Genotypes for controls			HWE test

					AA	AG	GG	AA	AG	GG	
Cui et al	2005	China	Asians	143/72	77	52	14	55	16	1	0.89
Liu et al	2005	China	Asians	76/60	46	27	3	47	12	1	0.82
Zhang et al	2006	China	Asians	94/68	55	39	0	57	11	0	0.46
Dai et al	2010	China	Asians	96/96	47	48	1	62	33	1	0.13
Sun et al	2010	China	Asians	91/42	67	24	0	33	9	0	0.44
Wu et al	2010	China	Asians	252/227	183	61	8	168	55	4	0.84
Zheng et al	2014	China	Asians	160/143	94	51	15	125	14	4	0.00

HWE, Hardy–Weinberg equilibrium

### Meta-analysis results

The major outcomes of our meta-analysis were shown in Figure 2. Q576R polymorphism was significantly correlated with elevated risk of pediatric asthma (GG vs. AA: OR = 3.75, 95% CI = 1.89–7.45; AG vs. AA: OR = 2.15, 95% CI = 1.36–3.39; the dominant model: OR = 2.25, 95% CI = 1.42–3.57; the recessive model: OR = 3.05, 95% CI = 1.54–6.05). The final result remained unchanged after eliminating the non-HWE research stratification analysis, suggesting the reliability and stability of our findings.

**Figure 2:**
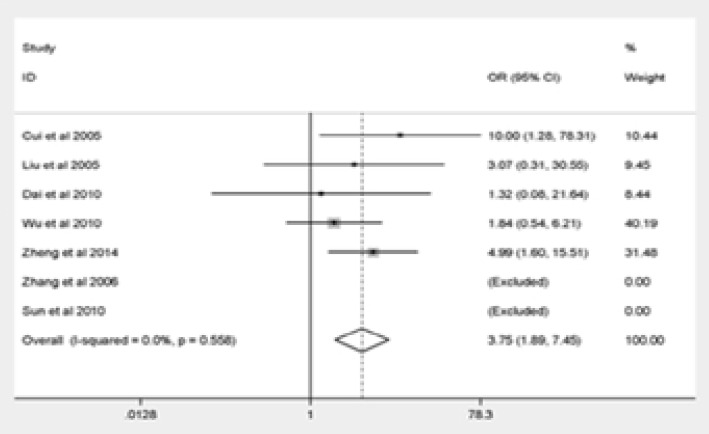
Forest plot for meta-analysis of the association between the IL-4R Q576R polymorphism and pediatric asthma risk(GG vs AA).

**Figure 2:**
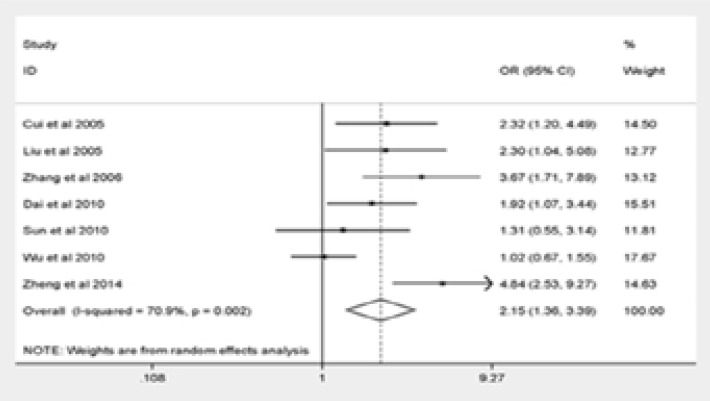
Forest plot for meta-analysis of the association between the IL-4R Q576R polymorphism and pediatric asthma risk(AG vs AA).

**Figure 2:**
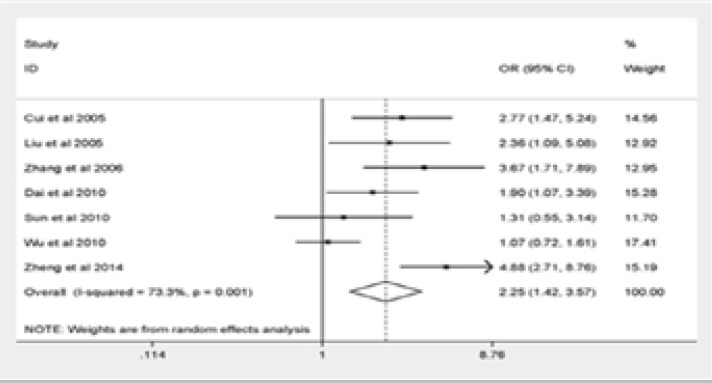
Forest plot for meta-analysis of the association between the IL-4R Q576R polymorphism and pediatric asthma risk(Dominant model).

**Figure 2:**
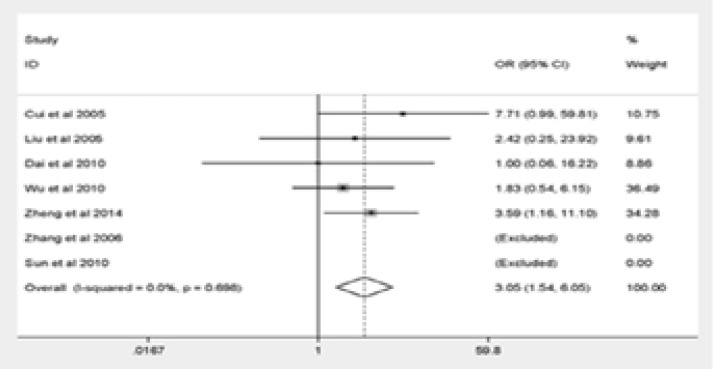
Forest plot for meta-analysis of the association between the IL-4R Q576R polymorphism and pediatric asthma risk(Recessive model).

### Publication bias

The Begg's test was performed for evaluating publication bias. Consequently, there no obvious evidence of publication bias by visually assessing funnel plot (Figure 3), implicating the low publication bias of our meta-analysis.

**Figure 3:**
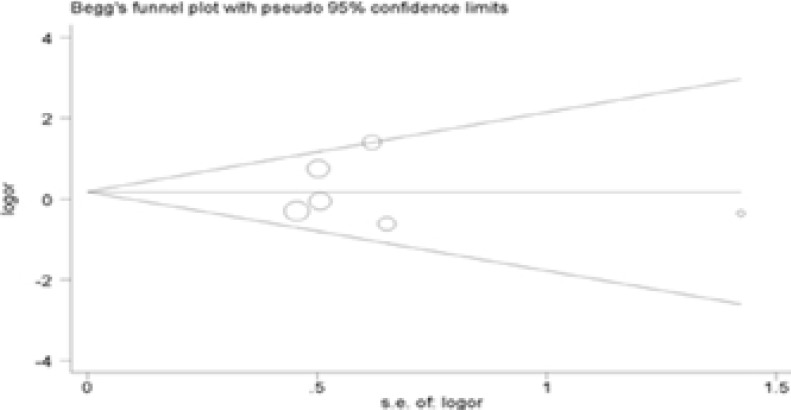
Begg's funnel plot analysis to detect potential publication bias for IL-4R Q576R polymorphism(GG vs AA).

**Figure 3:**
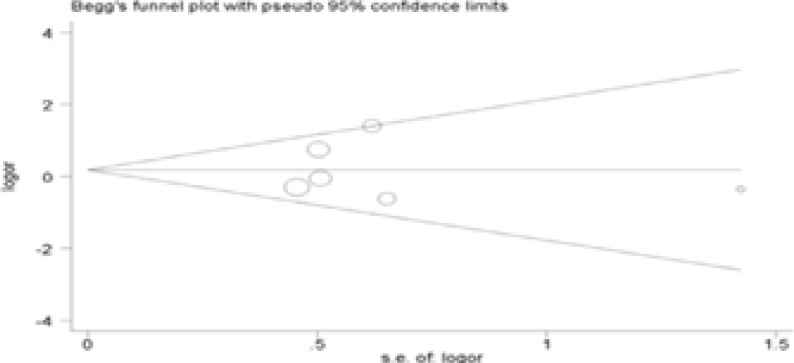
Begg's funnel plot analysis to detect potential publication bias for IL-4R Q576R polymorphism(AG vs AA).

**Figure 3:**
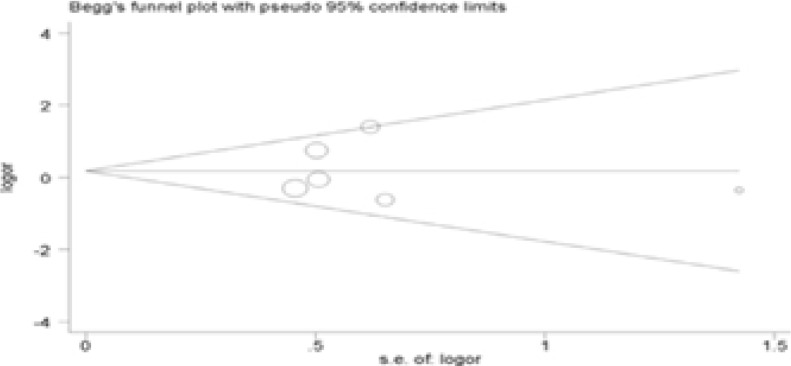
Begg's funnel plot analysis to detect potential publication bias for IL-4R Q576R polymorphism(Dominant model).

**Figure 3:**
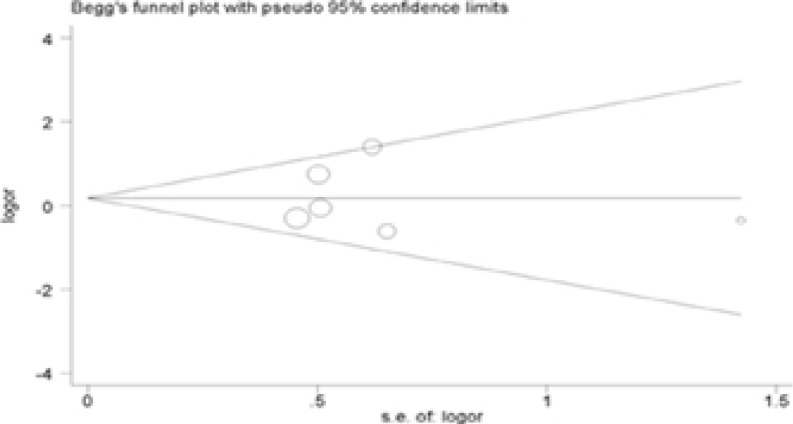
Begg's funnel plot analysis to detect potential publication bias for IL-4R Q576R polymorphism(Recessive model).

### Trial Sequential Analysis

**Figure 4:**
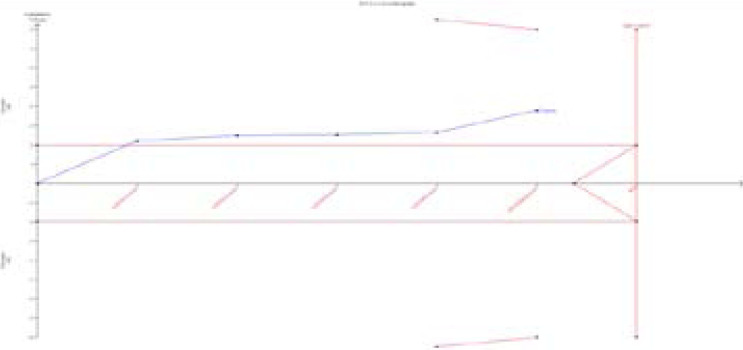
TSA for IL-4R Q576R polymorphism. We calculated a-spending adjusted required information size (RIS) by using a = 0.05 (two-sided), power = 80%. The cumulative Z-curve (Blue); Conventional boundary (Deep red); TSA boundary (red).

For reducing the random errors and increasing the credibility of the conclusions, TSA was performed. It showed that the cumulative Z-curves did not cross monitoring boundaries and futility boundaries and also did not reach the required information size (Figure 4). Thus, the results were potential false negative in additive models(GG vs. AA), and more studies were needed to conduct.

## Discussion

Asthma is considered as the most prevalent chronic pediatric disorder. The complicated pathogenesis of asthma remains largely unclear, despite multiple genetic loci as well as diverse environmental factors which are indicated to play decisive roles^26^. Genetic association study is considered as an effective way to determine asthma susceptibility associated with SNPs of candidate genes and has been widely applied in asthma research. However, the studies including inadequate samples may have weak statistical power and thereby interfere with the precision of results, leading to false positive or false negative findings consequently. Here, we performed a meta-analysis of published studies to evaluate the association between IL-4R Q576R polymorphism and risk of pediatric asthma. In conclusion, we found that IL-4R Q576R polymorphism might contribute to pediatric asthma risk.

Previous meta-analysis showed that IL-4R Q576R polymorphism is associated with asthma, included adults and children, but there was no subgroup analysis aiming at age in this article^27^. In the largest asthma GWAS to date, genetic risk loci for adult onset asthma is largely a subset of the loci associated with childhood onset asthma, with overall smaller effect sizes for onset at later ages8. Because childhood asthma is highly related to genes compared with adult asthma, we perform this meta-analysis for pediatric asthma. In conclusion, our findings showed that G allele of IL-4R Q576R polymorphism is associated with increased risk of pediatric asthma. In consideration of the possible between-study heterogeneity caused by deviation of allelic distributions from HWE, sensitivity analyses (only those consistent with HWE were analyzed by meta-analysis) were conducted, showing that the meta-analysis was reliable and realistic. Additionally, no evidence owed possible publication bias in our study.

Nevertheless, the mechanism underlying the relationship between IL-4R Q576R polymorphism and pediatric asthma risk remains unknown. To be specific, IL-4R-encoded protein is a vital functional component of Th2 cells. Previous study showed that The IL-4R Q576R polymorphism may involve in the development of allergy through modulating specific serum IgE levels^28^. While IL-4R-deficient mice cannot generate IgE and show defective Th2 responses, implicating that IL-4R plays a critical role in regulating IgE, and Q576R polymorphism could influence signal transduction, thereby elevating asthma risk.28 In addition, the possible effect of Q576R polymorphism could be influenced by gene-gene interaction. Previous study showed that IL-4-C33T, IL-13 R130Q, IL-4R I75V, IL-4R Q576R, STAT6 C2892T, and CD14-C159T may synergistically enhance the vulnerability to pediatric asthma^29^. Interaction between other risk factors and this polymorphism in relation to pediatric asthma should be further studied.

There were several limitations in our study. To begin with, based on unad¬justed information, we were unable to obtain genotype data after stratification for major confounding factors; additionally, there was great variation on confounding factors among different researches. Secondly, only seven articles from China were selected in the present meta-analysis. This may be one of the main limitations of the present work. Thirdly, with the merely published studies included in our meta-analysis, publication bias is very likely to occur, though no statistically significant publication bias is found in our meta-analysis. Fourth, age could be an important one, as the results for outcome onset before school age, before adolescence, and puberty can be different. Due to the small number of articles, we could not conduct a subgroup analysis for age. Finally, the predictive value of a single gene test in a complex disease is very limited for diagnostic or preventive purposes and thus cannot be recommended. Based on data of simulation studies and other complex diseases, the use of genetic profiling that incorporates multiple genetic risk factors holds promise for clinical application.

The results of genome-wide association studies will be crucial in establishing this genetic risk profile for asthma. In the future, asthma prediction may be possible, based on a prediction model that incorporates genes, personal factors and environmental risk factors^30^.

In conclusion, we demonstrate the significant relationship of IL-4R Q576R polymorphism with pediatric asthma. Population-based, large-scale, case-control studies should be performed to confirm the identified risk factors in our study, and to further explore other potential gene-environment and gene-gene interactions on pediatric asthma risk.
